# Evaluating Brazilian Agriculturalists’ IoT Smart Agriculture Adoption Barriers: Understanding Stakeholder Salience Prior to Launching an Innovation

**DOI:** 10.3390/s22186833

**Published:** 2022-09-09

**Authors:** Robert Strong, John Thomas Wynn, James R. Lindner, Karissa Palmer

**Affiliations:** 1 Department of Agricultural Leadership, Education, and Communications, Texas A&M University, College Station, TX 77843, USA; 2 Coastal Warehouse Limited, Wharton, TX 77488, USA; 3 Department of Curriculum and Teaching, Auburn University, Auburn, AL 36849, USA

**Keywords:** diffusion barriers, sustainability, Industry 4.0 technologies, agricultural innovation systems, knowledge transfer

## Abstract

The study sought to: (1) evaluate agriculturalists’ characteristics as adopters of IoT smart agriculture technologies, (2) evaluate traits fostering innovation adoption, (3) evaluate the cycle of IoT smart agriculture adoption, and, lastly, (4) discern attributes and barriers of information communication. Researchers utilized a survey design to develop an instrument composed of eight adoption constructs and one personal characteristic construct and distributed it to agriculturalists at an agricultural exposition in Rio Grande do Sul. Three-hundred-forty-four (*n* = 344) agriculturalists responded to the data collection instrument. Adopter characteristics of agriculturalists were educated, higher consciousness of social status, larger understanding of technology use, and more likely identified as opinion leaders in communities. Innovation traits advantageous to IoT adoption regarding smart agriculture innovations were: (a) simplistic, (b) easily communicated to a targeted audience, (c) socially accepted, and (d) larger degrees of functionality. Smart agriculture innovation’s elevated levels of observability and compatibility coupled with the innovation’s low complexity were the diffusion elements predicting agriculturalists’ adoption. Agriculturalists’ beliefs in barriers to adopting IoT innovations were excessive complexity and minimal compatibility. Practitioners or change agents should promote IoT smart agriculture technologies to opinion leaders, reduce the innovation’s complexity, and amplify educational opportunities for technologies. The existing sum of IoT smart agriculture adoption literature with stakeholders and actors is descriptive and limited, which constitutes this inquiry as unique.

## 1. Introduction

The technological pillars of the Industry 4.0 revolution include Internet of Things (IoT), machine learning (ML), artificial intelligence (AI), robots, drones, 5- and 6G systems, blockchain, virtual and augmented reality, and 3D printing [1]. Improving adoption of smart agricultural technologies is a focus of global institutions and organizations [2]. Agricultural extension’s success in disseminating information that promotes farmers’ adoption of contemporary agricultural technology is critical in the adoption cycle [3]. Kilelu, van der Lee, Koge, and Klerkx [4] identified that farmers’ minimal adoption of technology negatively affected the demand of Service Providers Enterprises. Knowledge and comprehension of an innovation are necessary for agriculturalists’ adoption and diffusion within their respective social systems [5,6]. Information and communication technologies (ICTs) provide indigenous information for marginalized farmers in developing countries [6]. The findings of Zambon et al. [7] revealed that, as Industry 4.0 has rapidly advanced and is approaching 5.0, Agriculture 4.0 is not as extensively adopted by stakeholders and is not widely examined by agricultural sciences scholars.

Agriculturalists in developing nations are prone to more production and technological barriers than peers in developed nations. Technological and information transmission are chief barriers experienced by agriculturalists [5]. Low education levels and inadequate communications contribute to a lack of adoption of smart agriculture innovations [6]. Some authors [8,9] suggested that optimum behavior change theories exist, but a favorable singular theory to frame an innovation adoption study does not exist for agriculturalists in developing nations. Agricultural extension change agents require better comprehension of innovation adoption components to improve professional development efficacy of the adoption cycle [10]. Barriers prohibiting farmers’ adoption of IoT smart agriculture technologies may be reduced when professional development experiences for agricultural extension personnel are offered.

Advancements in education have proven to change individual and organizational acceptance and use of newer technologies [11]. Extension programming that communicates the benefits of the innovation may motivate adoption in local communities and organizations, thereby improving the lives of individuals and communities. Farmer field schools are a proven outlet to increase agriculturalists’ knowledge respective to innovation’s advantages. Besides the improvement in knowledge, farmer field schools can benefit improving agriculturalists’ perceptions of new innovations in social learning environments aligning with agriculturalists’ social norms [12,13]. Additionally, educational experiences improve access to information and can increase marginalized agriculturalists’ awareness of IoT smart agriculture innovations. Using preexisting communications platforms, such as information communication technologies (ICTs), to educate and communicate innovations to agriculturalists has proven to expand knowledge transfer [14]. Concurrently, communications through existing accepted platforms increase agriculturalists’ knowledge without the peer pressure to make a decision quickly [15]. The internet and IoT innovations have materialized as practical educational technologies for agriculturalists in developing countries to improve their knowledge of innovations. Inadequate computers and minimal intellectual aptitude with technology are barriers to agriculturalists’ adoption of the internet as an avenue for knowledge transfer [16]. Developing comprehension of agriculturalists’ educational wishes and gaps addresses chief attributes of enhancing the adoption and dissemination of innovations in developing nations [17].

The theoretical framework implemented to assess the extent of IoT smart agriculture adoption and diffusion by Rio Grande do Sul agriculturalists was Rogers’ [18] diffusion of innovations. Diffusion of innovations is globally recognized and employed to examine technology and information adoption in diverse environments. Rogers’ [18] pivotal investigations posited the diffusion of innovations was functional to several fields of study, including education, business, medicine, and agriculture. Researchers have implemented diffusion as the scaffold to examine innovation adoption from various multidisciplinary fields. Interdisciplinary and transdisciplinary inquiries have been developed to ascertain diffusion theory’s validity in dissimilar environments. MacVaugh and Schiavone [19] reported that increased depth of innovation comprehension of diffusion was accomplished at various social system stages and subject areas. Information dissemination can be depicted by the diffusion of innovations at various macro and micro stages. The diffusion of innovations is investigated through the theory’s inclusion in local, national, and international communities [20]. Evaluating technology adoption has been assessed with diffusion of innovations previously. Hilbert [21] harnessed diffusion of innovations to assess the impacts of technology improvements on Latin American groups and potential outcome impacts.

Rogers [18] discerned five principal innovation characteristics that influence an innovation’s adoption and diffusion. The attributes promoting innovation adoption are the relative advantage of the innovation, its compatibility, the innovation’s complexity, trialability of the innovation, and the innovation’s visibility, which Rogers coined as observability. Relative advantage is a perceived advantage of the innovation prior to what is used [18]. An innovation’s relative advantage may be promoted or communicated from an environmental, economic, or societal lens to improve innovation adoption.

The extent an innovation is aligned with the subjective norms, needs, and values of agriculturalists is referred to as compatibility. When an innovation adheres to subsequent norms of the target audience, agriculturalists are more likely to adopt the innovation. Complexity refers to the innovation’s ease of understanding and applied use. Larger extents of or substantial variations in perceived complexity produce higher probabilities that an innovation will not be adopted.

The component linked to agriculturalists’ capacity to experiment with an innovation is called trialability. Rogers suggested opportunities to provide innovation trialability are predicated on utility of access, change agent and opinion leaders’ communication, and the innovation’s cost. Elevated trialability experiences produce increased likelihoods of innovation adoption. Increased experiences regarding innovation trialability produce higher likelihoods the innovation will be adopted by agriculturalists.

The extent to which agriculturalists can view traits of the innovation is the last attribute, referred to as observability. The more an innovation is observable, the higher the probability the innovation will be adopted within the social system [18].

Adopters’ categories are based on agriculturalists’ distinctive characteristics. Rogers [18] outlined five adopter categories, postulating the progressive shape of the diffusion ‘S’ curve. The adopter classifications developed in Ref. [18] were the innovators, then the early adopters, the early majority, the late majority, and, finally, the laggards. Venturesome is used to describe the innovators. The innovators more than likely had higher financial resources, a larger extent of intellectual astuteness, and were more apt to accept risk.

The highest level of social status in communities belongs to the early adopter members. The early adopters possessed higher intelligence and aversion to risk capacities. Early adopters tend to be older and more established than innovators. Early adopters are viewed as the most credible adopter classification in the social system [18].

Early majority adopters have similar characteristics to early adopters, with the principal difference that the early majority members did not have leadership roles (Strong et al.) [22]. Another dissimilarity is early majority members do not possess as much aversion to risk as innovators and early adopters within social systems.

The late majority classification was identified as having larger degrees of cynicism of the innovation’s advantages, but they were inclined to implement the innovation upon learning it was mandated or required in their agricultural operation. Late majority adopters likely own less or have less access to financial resources than the previous three categories and are more averse to risk.

Laggards was the name given to the fifth adopter classification. Laggards maintained traditional agricultural practices versus adopting contemporary innovations or techniques. Laggards have limited access to monetary funds, resulting in the highest aversion to risk compared to any other adopter category.

Additional scholars have examined individual acceptance of innovations. Goldberg [23] developed the five-factor model to describe the extent of the effect of an individual’s personality on their decision to adopt an innovation. Individuals’ personality characteristics elicit large amounts of persuasion on individual adoption [24,25]. Individuals chose to accept or reject innovations based on their perceived performance expectancy with the innovation, the effort expectancy with the innovation, social influences promoting the innovation, and facilitating conditions that provide organizational infrastructure for the innovation [26]. However, the authors want to be clear that agriculturalists’ personalities did not constitute the focus or an objective of our study [27].

The study was implemented to assess the extent of IoT smart agriculture adoption and diffusion by agriculturalists in the Brazilian province of Rio Grande do Sul. Our study’s objectives were: (1) to understand innovations’ characteristics of IoT smart agriculture that promote adoption and diffusion; (2) to describe agricultural IoT innovations’ adoption cycle; and (3) to discern the primary attributes of adoption of and resistance to IoT smart agriculture.

## 2. Materials and Methods

Descriptive and correlational paradigms were our study’s research design. The advantages of descriptive statistics are the illumination of participants’ beliefs, perspectives, and potential IoT smart agriculture innovations currently adopted [28]. Analyzing any potential association among variables, to better understand generalizability, was examined using correlational statistics [29]. Our multi-dimensional research design provided the inquiry of current construct discrepancies based on the data collected from agriculturalists in Rio Grande do Sul.

The population of agriculturalists in Rio Grande do Sul was 3289 at the time of the study. Authors examined 369 similar agriculturalists as a result of the simple random sampling technique. Data were gathered during meetings of agricultural cooperatives in Rio Grande do Sul. Researchers limited the sample size to agriculturalists who understood IoT smart agriculture innovations and approaches cooperatives took to market products, information, and technologies to members. Researchers omitted twenty-five incomplete surveys that were submitted, and, therefore, the final number in the data analysis was 344 agriculturalists [30].

Rio Grande do Sul was chosen as the province location due to the population characteristics of agriculturalists in the region. Agriculturalists were uniform and social economic status was not significantly different among the population. The agriculturalists lived primarily in rural areas but dispersed geographically in Rio Grande do Sul. Research and agricultural extension centers are utilized as the central hubs for research and sources of information. The research team based the IoT smart agriculture adoption instrument on Moore and Benbasat’s [31] investigating technology innovation adoption assessment. Agricultural professionals in Rio Grande do Sul in collaboration with Texas A&M University social science researchers and Auburn University researchers assessed content validity from four instrument iterations before the instrument was distributed to the population. Researchers concentrated on cultural sensitivity and awareness, the intentionality of the study, and improved the study’s clarity for the population. Global Speak Translations was used to translate the English instrument to Portuguese, as Portuguese is the indigenous Brazilian language, to improve response rate and reduce measurement error. Due to the English-first culture of the research team and the Portuguese-first language paradigm of the agriculturalist population, all items in the instrument were developed to reduce sampling error, coverage error, measurement error, and nonresponse error to improve agriculturalists’ discernment and enable participants to respond correctly [32].

An instrument composed of nine sections was implemented for the data collection. The instrument’s construction and supervision were advised by the tailored design method from Dillman et al. [32]. A personal demographics division and eight attitudinal variables were housed in the instrument. The instrument contained a summated scale with: 1 = strongly disagree, 2 = disagree, 3 = neither agree nor disagree, 4 = agree, and 5 = strongly agree in order to measure all attitudinal variables. Higher attitudinal scores equated with more positivity of IoT smart agriculture innovations. Voluntariness of IoT adoption, relative advantage of IoT smart agriculture, compatibility of IoT smart agriculture, image of IoT smart agriculture, IoT smart agriculture ease of use, IoT smart agriculture demonstrability, IoT smart agriculture visibility, and the trialability of IoT smart agriculture innovations were the attitudinal variables assessed in the instrument.

Voluntariness of adoption of the innovation was examined with four items. Nine items were used to measure IoT smart agriculture innovation’s relative advantage. Four items measured compatibility and five questions assessed the image construct. Four items examined demonstrability and eight statements assessed the ease-of-use variable. Five items were utilized to assess visibility and trialability. Participants’ age, level of education acquired, gender, and current profession were all the personal or demographic characteristics gathered in the study.

We pilot tested the questionnaire in Rio Grande do Sul with 33 agricultural individuals. Our pilot test examined the validity and reliability of the survey instrument. We revised the instrument based on the validity and reliability pilot data. Our team implemented Cronbach’s alpha coefficients to measure the internal consistency of all items within each respective construct. Cronbach theorized reliability coefficients are a social science indicator for assessing the extent an instrument will produce consistent results [33]. Cronbach’s alpha coefficients provide an interconnected mean among items within an instrument [34]. In the social sciences, alpha coefficients greater than 0.07 are the lowest threshold for instrument reliability, with 1 as the highest coefficient threshold [35]. Rogers’ relative advantage yielded the highest internal consistency with a 0.90, then compatibility at 0.86, complexity produced a 0.84, the innovation’s visibility was 0.83, voluntariness was 0.82, demonstrability yielded a 0.79, also a 0.79 for the innovations image, and the trialability construct produced a coefficient of 0.71 [36].

SPSS 27 was used to analyze data collected. An alpha level of 0.05 significance was instituted *a priori* [33,34]. Dispersion of the data was measured using central tendency and standard deviations [36]. Frequencies and categorical data were utilized to illustrate categorical data. Authors implemented a regression analysis to describe interactions among variables. To measure the associations between variables, the authors employed a stepwise regression model [33]. The variance among constructs was examined using principal component analyses to describe, explain, and enhance interpretation of the data [35]. The variables were analyzed using summated scales. Summated scales offered an analytical foundation to discern and develop implications for the previously identified variables.

The differences, statistically, were analyzed among late and early participant responders to ensure the study’s external validity [37]. The authors implemented an ANOVA and *t*-tests to analyze the differences among late and early participant responders. There were no statistical discrepancies among early and late participant respondents. Outlined by Lindner et al. [37], our data are generalizable to the population represented in the study.

## 3. Results

Our initial objective was to understand the innovation characteristics of IoT smart agriculture that promote adoption and diffusion in the province. Agriculturalists’ individual attributes were assessed to understand the innovation and the innovation’s adoption characteristics. In order to understand the innovation’s characteristics, agriculturalists’ personal characteristics and responses of agriculturalists were used to identify innovative traits. Mean scores above 4.0 were representative of positive innovation traits. Scores ranging from 2 to 3.9 illustrated less advantageous or neutral innovation traits. Innovation elements with mean scores < 1.9 represented negative traits that would prohibit adoption or slow down the adoption cycle. Each attribute’s weight was evaluated by frequency statistics. The eight variables included in the instrument were examined for desirable, neutral, and undesirable traits, leading to adoption and diffusion or rejection.

Voluntariness’ traits of adoption was the first variable we measured. Expectation of use, voluntariness of use, mandated use, and the usefulness perceptions of IoT smart agriculture technologies were assessed by four items. The authors reverse coded items “I am not required to use technology as part of my job”, including “Technology is helpful performing my job” to reduce agreement absentmindedness issues identified by Dillman et al. Agriculturalists’ reported use expectation produced the largest (*M *= 4.34, *SD *= 0.55) voluntariness inspiration in the adoption cycle illustrated in Table 1. Positive adoption traits included required use, voluntary use, and the innovation’s perceived use due to mean scores over 4.0. Required use yielded the strongest weight of influencing an innovation’s adoption by more than 95% of agriculturalists. A vast majority of agriculturalists (97%) reported the anticipation of using the innovation contributed to the adoption, implying individual objectives and social influence were predictive indicators of voluntariness. The grand mean was 4.29 (*SD* = 0.53) and Cronbach’s alpha was 0.77. The anchors to assess voluntariness were: 1 = Strongly Disagree, 2 = Disagree, 3 = Neither Agree nor Disagree, 4 = Agree, and 5 = Strongly Agree.

The perceptions of achieving objectives quicker, developing premium products, making life routines simple, easier daily work goals, improving professional outcomes, producing higher-quality staff, increased individual agility, and enhanced agricultural productivity were the items used to assess IoT smart agriculture’s relative advantage. The item earning the highest mean score (*M* = 4.42, *SD* = 0.61) was easier daily work goals. Items earning mean scores > 4.0 indicating positive attributes included IoT smart agriculture that achieves objectives quicker, improving professional outcomes, enhancing agricultural productivity, producing higher-quality staff, and increasing individual agility. Making life routines simpler earned the lowest mean (*M* = 3.35, *SD* = 1.22), deemed a neutral versus a positive characteristic. Researchers examined the influence of the neutrality by excluding making life routines simpler from another round of Cronbach’s alpha analysis to determine the reliability coefficient. The majority of agriculturalists (93%) believed IoT smart agriculture innovations rendered them an easier lifestyle. The grand mean for relative advantage was *M* = 4.19, *SD* = 0.87, and 0.82 was the reliability coefficient. Attributes were measured with anchors: 1 = Strongly Disagree, 2 = Disagree, 3 = Neither Agree nor Disagree, 4 = Agree, and 5 = Strongly Agree (see Table 2).

Compatibility was examined from agriculturalists’ responses to items representative of the extent specific innovations were recognized as congruent with work mandates, augment current work techniques, align with work expectations, and align with lifestyle. The highest compatibility mean score (*M *= 3.94, *SD *= 0.74) was produced by a capacity to align with agriculturalists’ work expectations. Mean scores for each assessed attribute earned scores from 2.0 to 3.90 as almost 80% of agriculturalists reported agreement or strong agreement. Data indicated IoT smart agriculture innovation compatibility traits were neutral statistically or not as suitable for adoption. The capacity of an innovation to align with agriculturalists’ work expectations and the capability of incorporation in their daily lifestyle was reported by 70% of agriculturalists. Compatibility’s grand mean was 3.85 (*SD* = 0.8) and the reliability coefficient was 0.82 (see Table 3). The scale was measured with: 1 = Strongly Disagree, 2 = Disagree, 3 = Neither Agree nor Disagree, 4 = Agree, 5 = Strongly Agree.

In order to assess image, four attributes were asked of agriculturalists: IoTs improve an individual’s image, agriculturalists acquire more value in society, increased recognition from social systems, and if IoTs are representations of status. IoTs improve an individual’s image was the highest attribute (*M* = 3.79, *SD* = 0.72) on agriculturalists’ decision to adopt IoT smart agriculture technology. Next, agriculturalists acquire more value in society (*M* = 3.35, *SD* = 0.94) earned the second highest mean. Based on mean scores (between 3.11 and 3.79), the data indicated image attributes were neutral at best. The belief IoT smart agriculture adoption provided a liftoff for agriculturalists’ image was accepted by 67% of the agriculturalists participating in our study. More than 50% of agriculturalists did not respond positively to IoT smart agriculture enhancing peers of society’s judgement as a whole of agriculturalists, including their social status. Image’s grand mean was 3.41 (*SD* = 0.96) and image’s reliability coefficient was 0.81 (see Table 4). The instrument anchors were: 1 = Strongly Disagree, 2 = Disagree, 3 = Neither Agree nor Disagree, 4 = Agree, 5 = Strongly Agree.

Agriculturalists answered individual ease of use attribute items. Measureable attributes included transparency of IoT smart agriculture usage, usage procedures are easy to cognitively retain, IoT smart agriculture technologies are simple to learn, IoT smart agriculture technologies are manageable, IoT smart agriculture technologies usage is easy, IoT smart agriculture technologies usage is not difficult, IoT smart agriculture technologies are dependable, and, lastly, IoT smart agriculture technologies usage is routine. Transparency of IoT smart agriculture usage had the highest mean score (*M *= 3.87, *SD *= 0.68) and usage procedures are easy to cognitively retain (*M *= 3.83, *SD *= 0.77) was next. Each ease of use attribute was assessed to be the most neutral and at the least not containing desired innovation traits, and 75% of agriculturalists reported transparency of IoT smart agriculture use was important to them. Data indicated use consistency and the stable ease of use had the lowest levels of importance due to < 50% of agriculturalists reporting *strongly agree* or *agree*. Ease of use’s grand mean was 3.57 (*SD* = 0.89) and yielded a reliability coefficient of 0.92 (see Table 5). The survey anchors included: 1 = Strongly Disagree, 2 = Disagree, 3 = Neither Agree nor Disagree, 4 = Agree, 5 = Strongly Agree.

Demonstrability, the sixth dissemination attribute variable, was assessed by attributes: prompt identifiable outcomes, communication easiness to social system peers, demonstration easiness to social system peers, and identifiable advantages for agriculturalists. Agriculturalists reported prompt identifiable outcomes as the chief demonstratability characteristic (*M* = 3.92, *SD* = 0.71) due to 79% indicated agreement to strong agreement. All demonstratability trait characteristics were neutral or non-desirable. The least impactful attribute (*M* = 3.39, *SD* = 0.95) was identifiable advantages for agriculturalists as < 54% of agriculturalists reported some level of agreement. Demonstratiability earned a grand mean of 3.77 (*SD* = 0.81) and a 0.88 for reliability coefficient (see Table 6). The attribute anchors were: *1 =* Strongly Disagree, 2 = Disagree, 3 = Neither Agree nor Disagree, 4 = Agree, 5 = Strongly Agree.

Visibility was measured by four attributes. The adoption attributes were social system use of IoT smart agriculture is observable, noticeable in social system’s agricultural operations, clear flexibility of IoT smart agriculture innovations, and IoT smart agriculture usage in the community. The highest scoring mean was social system use of IoT smart agriculture is observable (*M* = 4.16, *SD* = 0.63) and 90% of agriculturalists agreed to strongly agreed. Agriculturalists further reported agreement of IoT smart agriculture innovations noticeable in social system’s agricultural operations (*M* = 4.07, *SD* = 0.66). Both clear flexibility of IoT smart agriculture innovations and IoT smart agriculture usage in the community were neutral or non-desirable characteristics. The grand mean for visibility was 3.96 (*SD* = 0.71) and the construct produced a reliability coefficient of 0.94 (see Table 7). The visibility anchors were: 1 = Strongly Disagree, 2 = Disagree, 3 = Neither Agree nor Disagree, 4 = Agree, 5 = Strongly Agree.

Trialability was our last variable to assess and was measured using five attributes. The characteristics included the easiness of finding new IoT smart agriculture technologies, new experiences with IoT smart agriculture technologies, adequate to experiment with IoT smart agriculture technologies, convenience of IoT smart agriculture technologies usage, and opportunities to test IoT smart agriculture technologies. Indeed, 62% of agriculturalists reported some level of agreement with the easiness of finding new IoT smart agriculture technologies (*M* = 3.67, *SD* = 0.89). Agriculturalists reported opportunities for new experiences with IoT smart agriculture technologies met trialability metrics (*M* = 3.56, *SD* = 0.92). Data indicated all trialability attributes were not positive. Agriculturalists reported < 50% agreed the timeframe to acquire and test IoT smart agriculture was adequate. Trialability’s grand mean was 3.41 (*SD* = 0.94) and the construct produced a reliability coefficient of 0.70 (see Table 8). The scale anchors were: 1 = Strongly Disagree, 2 = Disagree, 3 = Neither Agree nor Disagree, 4 = Agree, 5 = Strongly Agree.

Our third objective sought to identify IoT smart agriculture’s adoption cycle by Rio Grande do Sul agriculturalists. The researchers categorized agriculturalists into adopter categories: (1) innovators, (2) early adopters, (3) early majority, (4) late majority, and (5) laggards based on the results from responses to the eight adoption variables. The researchers sought to comprehend agriculturalists’ characteristics specific to each individual classification. Adopting IoT smart agriculture voluntarily was used to determine voluntariness. IoT smart agriculture offering opportunities for simpler work was the innovation’s relative advantage, and the extent agriculturalists incorporated IoT smart agriculture into their agricultural operation indicated compatibility. Status symbol responses were utilized to determine image, and IoTs manageable characteristics were employed to answer easiness of usage. Demonstrability and visibility were assessed using the promptness of IoT smart agriculture outcomes and the simpleness of observable use in communities. Agriculturalists’ capacity to experiment with IoT smart agriculture technologies was implemented to measure trialability. Innovators were assigned to those reporting strong agreement to all eight variables. Early adopters were designated to agriculturalists responding with agreement to strong agreement but removing innovator responses. The early majority was determined by examining agriculturalists responding neither agree or disagree, agree, or strongly agree in the eight constructs but not previously categorized as early adopters or innovators. Agriculturalists were identified in the late majority if responding strongly agree, agree, neither agree or disagree, and disagree and not initially assigned to the innovator, early adopter, or early majority classifications. Seventeen agriculturalists perceived themselves as laggards and one agriculturalist reported being an innovator (see Table 9).

At the conclusion of the adopter category analyses, the researchers implemented a stepwise regression model. The purpose of the stepwise regression model was to categorize IoT smart agriculture adopters from examining each variable attribute. Our independent or antecedent variables were the voluntariness of the innovation, IoTs’ relative advantage, IoTs’ compatibility, agriculturalists’ image from adoption of IoTs, IoTs’ easiness of usage, IoTs’ demonstrability, the visibility of IoTs, and the extent IoTs were trialable. The dependent variable was adopter classifications.

The stepwise regression findings revealed, out of 45 items, nine attributes described 63.8% of adoption variance. Antecedent variables are delineated in Table 10. Data from the stepwise regression indicated adoption was enlarged as agriculturalists’ pressure from peers also increased. The robustness of attitudes regarding IoT smart agriculture enhanced productivity, and higher levels of adopter classifications regarding life routines were achieved. The individual’s work, the perception of using technology as a status symbol, the perception that technology is easy to manipulate, observing others in the community using technology, the ability to easily communicate technology, and the ability to properly test a new innovation were found to enhance the adoption process. Agriculturalists reporting challenges in feeling the initial impact of IoT smart agriculture was an indicator of those individuals moving down the adopter classifications, resulting in nonadoption. Earning statistically significance were demonstrability, agriculturalists’ image, IoTs’ voluntary adoption, IoT smart agriculture observability, and opportunities for IoT trialability with *p* < 0.05. Not statistically significant indicators of adopter classifications were IoTs’ relative advantage, IoTs’ compatibility, and IoTs’ easiness of use. The stepwise regression model produced R^2^ = 0.64, *F* = 43.11.

Our study’s fourth aim sought to describe primary characteristics and IoT smart agriculture resistors precluding innovation adoption in Rio Grande do Sul. A stepwise regression was employed to assess innovation rejection attributes predicting knowledge transfer barriers. The researchers included dummy variables. Non-adopters were denoted with a zero value and adopters were identified with one as their numerical value. All attributes within our instrument were employed as antecedent variables in the stepwise regression. The significant attributes were utilized to describe characteristics of IoT smart agriculture innovations impacting rate of adoption. Table 11 illustrates the SPSS version 27 output of voluntariness use of IoTs, the relative advantage of IoTs, IoTs’ compatibility, agriculturalists’ image, and IoTs’ easiness of use.

The model described 41.6% in adopter categories variance. IoT smart agriculture technololgies improving social status, were not challenging to use, were easily observable, and offered an adequate timeframe for agriculturalists to test positively influenced stakeholder adoption and diffusion. On the contrary, IoT smart agriculture innovations solely making routibnes easier, not easily manageable, not perceived as increasing performance, and were complex to understand were not indicative of IoT smart agriculture adoption or diffusion. As one unit increased, the probability of adoption increased. As a unit of my social system wants me using IoT smart agriculture technology increased by 0.04, the probability of IoT smart agriculture technology adoption improved. The regression model produced R^2^ = 0.42, *F* = 14.93 and * p < 0.05.

## 4. Discussion

Communication methods, strategies, and accessibility are not keeping pace with the exponential rate that new IoT smart agriculture technologies are being introduced. Improving communication rates, outlets, and accessibility is paramount to increase the rate of adoption as future multifaceted IoT smart agriculture innovations increase with advancements in technology [5,6]. If conventional communication channels do not have the capability to successfully communicate the advantageous innovation attributes to agriculturalists, then the low rate of adoption will persist. Based on Rogers [18], in a social system, change agents should emphasize relationship building, development, and communication of opinion leaders to improve the communication of positive attributes of IoT smart agriculture to stakeholders in order to enhance the rate of adoption. We recommend information dissemination for the agriculturalist be aligned with the innovation’s characteristics and the targeted audience goals and individual characteristics.

Complex IoT smart agriculture futures markets exhibited a reduced frequency of engagement in the population. This study revealed profits from IoT smart agriculture adoption served as a relative advantage for agriculturalists and would enhance adoption over potential risks by providing economic incentives [38]. Reducing the innovation’s complexity coupled with purposeful and succinct communication of the IoT smart agriculture technology’s advantages to agriculturalists and opinion leaders will improve adoption and diffusion within the social system. Our data indicated targeting communications at agriculturalists’ work location and in localized communities would offer the maximum successful strategies to disseminate IoT smart agriculture information. It is imperative for practitioners to communicate the benefits of IoT smart agriculture technologies to agricultural owners and managers, as well as community leaders, to advance the livelihoods and sustainability of individuals and resulting community impacts. Communications from researchers to practitioners and practitioners to agriculturalists have to be distinct and succinct for the adoption cycle and diffusion of IoT smart agriculture technologies [39]. Organizations promoting IoT smart agriculture ought to have the innovations promptly accessible for agriculturalists as soon as individuals have decided to test the technology per the trialability stage to improve the rate of adoption [18]. Avenues exist to increase IoT smart agriculture adoption, such as farmer field schools, the Ministry of Agriculture and institutional research stations in local communities, vocational education at local agricultural colleges, and agricultural field days and expositions [40].

To improve IoT smart agriculture adoption, we recommend newer innovations be visually appealing to stakeholders. As identified by Rogers [18], practitioner comprehension of social status and pressure from peers are critical for enhancing the adoption rate of early and late majority agriculturalists. The data indicated that resources for promoting IoT smart agriculture adoption ought to be understandable, practical, and offer client-oriented technical advice for the targeted audience so as to reduce the innovation’s complexity beliefs [41]. Ideally, IoT smart agriculture innovations would be diverse in application and functionality for agriculturalists.

The data did not specify educational discrepancies between agricultural adopters and resistors. Both adopters and rejectors of the innovation had attained equal levels of education. We recommend that change agents striving to promote successful adoption and diffusion of IoT smart agriculture emphasize enriching the innovation’s communication and purposely training the target audiences, resulting in progressively influencing IoT smart agriculture’s adoption [11].

Additional Brazilian smart agriculture adoption and diffusion research questions were revealed throughout the study, warranting future examination. Inquiries centered on diffusion in developing nations are limited in number and breadth. We recommend replicating our study in other regions of the world to increase our collective understanding of IoT smart agriculture stakeholders, adoption characteristics, and the resulting impacts of IoT adoption.

The impact of opinion leadership during the adoption cycle was statistically significant on the prospect of adoption and diffusion. The greater the social status bestowed to the innovation’s adoption, the more this influenced IoT smart agriculture adoption in our study [18]. Rejectors of innovations bequeath trust on adopters and opinion leaders based on their decisions, but the level trust is not necessarily a tipping point characteristic for rejectors to move to the title of adopter. However, measuring trust and the resulting variance trust sways in the adoption cycle, from an unequivocal paradigm, is challenging to ascertain. Discerning the significance of trust as a construct that predicts or influences the adoption cycle is worthy of additional investigation. Future studies should assess techniques or approaches that change agents could employ to positively promote IoT smart agriculture adoption among stakeholders.

As one of the largest agricultural producers in the Western Hemisphere, understanding Brazilian agriculturalists’ adoption and rejection of innovations designed to improve food security and sustainability [42] is a necessity given agriculturalists’ impact locally, nationally, and across the continent and the world. Extra analysis into the IoT smart agriculture adoption cycle is necessary throughout Brazil and South America to assess innovation traits and personal characteristics of adopters that potentially promote adoption and diffusion. The supplementary data would expand our body of knowledge and potentially allow generalizability on a much larger scale [6].

Our study demonstrated the dissimilarity among age between adopters and those rejecting the IoT smart agriculture innovations. The literature indicated more training and educational opportunities for agriculturalists enhances their acceptance of the latest technologies regardless of the individual’s age [5]. Additional inquiries are needed to investigate impactful communication channels improving the communication of smart agriculture’s positive characteristics from researchers to practitioners and then practitioners to agriculturalists.

Societal influences and social status should not be disrespected in the adoption and diffusion IoT smart agriculture process. Both attributes were common denominators that floated to the top of the analysis and were illuminated throughout the study. We found escalating agriculturalists’ awareness and knowledge of IoT smart agriculture as an innovation boosted the adoption of the innovation per the adoption cycle, as identified in Ref. [18]. Auxiliary examinations of the distinctive characteristics of opinion leaders, change agents, and innovators should be performed to assist researchers’ and practitioners’ comprehension respective to agriculturalists’ social dynamics that foster the IoT smart agriculture adoption and diffusion process [34].

It is important to the inquiry’s field to develop the capacity of IoT smart agriculture adoption rate and the innovation’s traits improving adoption and diffusion. Experimental designs and longitudinal studies are necessary to measure potential associations between characteristics of the innovation, categories of adopters, and an innovation’s rate of adoption. The authors recommend similar investigations for virtual reality, artificial intelligence, and additional technology innovations. More robust comprehension of potential affiliations and linkages would enhance researchers’ and practitioners’ knowledge of attributes that predict IoT smart agriculture, virtual reality, artificial intelligence, innovation systems, and other technological innovations’ adoption and stakeholder diffusion.

## 5. Conclusions and Future Work

Agriculturalists’ adoption is predicted by their belief in IoT smart agriculture as providing a relative advantage, compatible with their existing needs, not challenging to use, easily testable to examine how to use the innovations, and possessing easily discernible benefits. Individuals’ adopter categories within the social system also affect adoption and diffusion of the IoT innovations. Our data highlighted that opinion leaders’ influence on the rate of adoption is essential for IoT researchers and practitioners in local communities to understand. The data supported Rogers’ research that rate of adoption and subsequent diffusion is maximized by inclusion communications with opinion leaders and utilizing their trust within the community to foster IoT smart agriculture adoption. The studies by Goldberg and McCrae and Costa Jr. on the five traits warrant inclusion in future IoT smart agriculture adoption inquiries. Globally, we need to better understand and predict social science decisions and impacts from adopted IoT, virtual reality, artificial intelligence, machine learning [43] and in every technical facet of agricultural innovation systems. Moving the needle forward for Agriculture 4.0 and eventually 5.0 in terms of innovation adoption and diffusion is essential to understand given global issues that include climate change, food security, water, sustainability, nutrition, and improving the lives of marginalized citizens across our world.

Future plans are to implement randomized control trials with adopters as the treatment and non-adopters or resistors of IoT smart agriculture as the control to better understand resulting individual, community, economic, social, and environmentally sustainable impacts due to innovation adoption. Our expansion of this inquiry is continuing in Africa and Northern Europe. Tremendous gains have been made in technology development and technology efficiency. However, as our data reported, innovations are only valuable to the extent that stakeholders choose to use them to advance their goals.

## Figures and Tables

**Table 1:** Voluntariness Innovation Metrics (*n* = 334).

Attributes	*M*	*SD*
Use expectation	4.39	0.52
Voluntariness of use	4.32	0.48
Usefulness perceptions	4.29	0.50
Mandated use	4.27	0.51

**Table 2:** Relative Advantage’s Innovation Metrics (*n* = 334).

Attributes	*M*	*SD*
Easier daily work goals	4.42	0.61
Develop premium products	4.41	0.66
Achieving objectives quicker	4.39	0.68
Improve professional outcomes	4.33	0.62
Enhanced agricultural productivity	4.29	0.64
Producing higher-quality staff	4.27	0.61
Increased individual agility	4.19	0.61
Makes life routine taskss simpler	3.35	1.22

**Table 3:** Compatibility’s Innovation Metrics (*n* = 334).

Attributes	*M*	*SD*
Aligns with work expectations	3.94	0.74
Augments work techniques	3.89	0.78
Aligns with lifestyle	3.86	0.79
Congruent with work mandates	3.69	0.91

**Table 4:** Beliefs of Agriculturalists’ Image due to IoT Smart Agriculture (*n* = 334).

Attributes	*M*	*SD*
IoT improves individual’s image	3.79	0.72
Agriculturalists acquire more value in society	3.35	0.94
Increased recognition from social systems	3.21	0.91
IoTs are representations of status	3.11	0.95

**Table 5:** Ease of Use’s Innovation Metrics (*n* = 334).

Attributes	*M*	*SD*
Transparency of IoT smart agriculture usage	3.87	0.68
Usage procedures are easy to cognitively retain	3.83	0.77
IoT smart agriculture technologies are simple to learn	3.79	0.77
IoT smart agriculture technologies are manageable	3.75	0.79
IoT smart agriculture technologies usage is easy	3.73	0.81
IoT smart agriculture technologies usage is not difficult	3.31	1.00
IoT smart agriculture technologies are dependable	3.24	0.93
IoT smart agriculture technologies usage is routine	3.18	0.96

**Table 6:** Demonstrability’s Innovation Metrics (*n* = 334).

Attributes	*M*	*SD*
Prompt identifiable outcomes	3.92	0.71
Communication easiness to social system peers	3.83	0.78
Demonstration easiness to social system peers	3.75	0.80
Identifiable advantages for agriculturalists	3.39	0.95

**Table 7:** Visibility’s Innovation Metrics (*n* = 334).

Attributes	*M*	*SD*
Social system use of IoT smart agriculture is observable	4.16	0.63
Noticeable in social system’s agricultural operations	4.07	0.66
Clear flexibility of IoT smart agriculture innovations	3.99	0.68
IoT smart agriculture usage in the community	3.69	0.79

**Table 8:** Trialability’s Innovation Metrics (*n* = 334).

Attributes	*M*	*SD*
The easiness of finding new IoT smart agriculture technologies	3.67	0.89
New experiences with IoT smart agriculture technologies	3.56	0.92
Adequate to experiment with IoT smart agriculture technologies	3.41	0.91
Convenience of IoT smart agriculture technologies’ usage	3.37	0.94
Opportunities to test IoT smart agriculture technologies	3.31	0.93

**Table 9:** Metrics of Agriculturalists’ Adopter Classifications (*n* = 294).

Adopter Categories	*f*	*%*
Late majority	135	40.4
Early majority	101	30.2
Early Adopters	40	12.0
Laggards	17	5.1
Innovators	1	0.3

**Table 10:** IoT Smart Agriculture Adoption Constructs.

Antecedent Constructs	*Beta*	*t*	*p*
The social system wants me to use IoT smart agriculture technologies	−0.96	−2.30	0.01 *
IoT smart agriculture technologies usage improves productivity	−0.01	−0.09	0.87
IoT smart agriculture technologies align with my agricultural routines	−0.02	−0.33	0.79
IoT smart agriculture technologies are compatible with agricultural operations	−0.08	−1.60	0.10
IoT smart agriculture technologies are community status symbols	−0.54	−12.67	0.00 *
IoT smart agriculture technologies are simple to manage	−0.07	−1.60	0.09
IoT smart agriculture technologies are easily communicable	0.11	2.45	0.00 *
My community’s IoT smart agriculture technologies are easily observable	−0.23	−5.21	0.00 *
IoT smart agriculture technologies were tested before I used them	−0.30	−6.36	0.00 *

* Note: *p* < 0.05.

**Table 11:** IoT Smart Agriculture Adoption Attributes.

Antecedent Constructs	*Beta*	*t*	*p*
My social system wants me using IoT smart agriculture technology	0.04	1.64	0.18
IoT smart agriculture technology makes me more productive	0.03	0.86	0.48
IoT smart agriculture technology makes my routine easier	0.00	−1.29	0.17
IoT smart agriculture technology aligns with my work patterns	−0.01	−0.48	0.65
Having IoT smart agriculture technology increases my status	0.08	6.13	0.00 *
Learning IoT smart agriculture technology is simple	0.06	1.97	0.09
IoT smart agriculture technology is challenging	−0.04	−2.34	0.00 *
IoT smart agriculture technologies are simple to control	0.01	0.92	0.46
IoT smart agriculture technologies are easily observable in my community	0.06	3.31	0.00 *
The timeframe to try out IoT smart agriculture technologies is adequate	0.12	5.39	0.00 *

* Note: *p* < 0.05.

## Data Availability

Data were collected in person and from card copies of the instrument. Data sharing is not applicable to this article.
